# Dataset of anaerobic acidogenic digestion for hydrogen production using xylose as substrate: Biogas production and metagenomic data

**DOI:** 10.1016/j.dib.2019.104466

**Published:** 2019-08-31

**Authors:** Gustavo Mockaitis, Guillaume Bruant, Serge R. Guiot, Eugenio Foresti, Marcelo Zaiat

**Affiliations:** a Interdisciplinary Research Group of Biotechnology Applied to the Agriculture and Environment, School of Agricultural Engineering, University of Campinas (GBMA/FEAGRI/UNICAMP), 501 Cândido Rondon Avenue, CEP 13.083-875, Campinas, SP, Brazil; b Anaerobic Technologies and Bioprocess Control Group, Energy, Mining and Environment Portfolio, National Research Council Canada, 6100 Royalmount Avenue, H4P 2R2, Montreal, QC, Canada; c Hydraulics and Sanitation Department, São Carlos Engineering School, Universidade de São Paulo (SHS/EESC/USP), 400 Trabalhador São-Carlense Avenue, CEP 13.566-590, São Carlos, SP, Brazil

**Keywords:** Dark fermentation, Hydrogen, Anaerobic digestion, Biogas, Xylose

## Abstract

This paper presents the raw data of biogas production and composition (relative pressures and concentrations of each of the biogas constituents) for batch experiments to evaluate the anaerobic digestion of xylose. Also, metagenomic sequencing data and analysis were reported. All data is available at Mendeley Data. 16S DNA sequencing data and metadata is available at MG-RAST (metagenomics.anl.gov/linkin.cgi?project = 9961). For further discussion, please refer to the scientific article entitled "Effect of acidic and thermal pretreatments on a microbial inoculum for hydrogen and volatile fatty acids production through xylose anaerobic acidogenic metabolism" (Mockaitis et al., 2020).

**Table:** Specifications Table

Subject area	*Biotechnology*
More specific subject area	*Bioenergy and biofuels*
Type of data	*Tables and graphs, Excel spreadsheets, FASTA sequences archives*
How data was acquired	*Desktop computer, manometer, gas chromatography (using a Shimadzu GC2010) and 16S DNA sequencing (using a Roche 454 Genome Sequencer FLX)*
Data format	*Raw data*
Experimental factors	*Hydrogen production, methane production, volatile organic acids concentrations, 16S DNA*
Experimental features	*Simple batch assays of 5 different inoculum pretreatments to improve hydrogen production through anaerobic digestion of xylose.*
Data source location	*São Carlos, SP, Brazil*
Data accessibility	*All data is available at Mendeley Data. (doi.org/10.17632/7knhxgvb4s.1). 16S DNA sequencing data and metadata is available at MG-RAST (metagenomics.anl.gov/linkin.cgi?project=9961)*
Related research article	Mockaitis, G.; Bruant, G.; Guiot, S.R.; Peixoto, G.; Foresti, E.; Zaiat, M. Effect of acidic and thermal pretreatments on a microbial inoculum for hydrogen and volatile fatty acids production through xylose anaerobic acidogenic metabolism. Renew Energ. 145, 1388–1398, 2020. https://doi.org/10.1016/j.renene.2019.06.134

**Table:** 

**Value of the data** • Biogas production and composition parameters are relevant to determine the viability of anaerobic digestion and are useful for further mathematical modeling and machine learning techniques for predicting the behavior of anaerobic bioreactors • Metagenomics data of pretreatments on anaerobic digestion inoculum provides an overview about microbial communities and is one of the major parameters to optimize the production of added-value products from anaerobic digestion • All data presented in this paper can be used as a source for comparisons with other studies in anaerobic digestion aiming hydrogen production

## 1 Data

Biogas production and composition was assessed for the control assay (Table 1) and the four different pretreatments performed in the original inoculum. Acidic pretreatment (Table 2), thermal (Table 3), acidic-thermal (Table 4), and thermal-acidic (Table 5) were evaluated considering the total relative pressure and concentration of H_2_, N_2_, CH_4_, and CO_2_. All raw data presented in this paper, including the sequences (in FASTA format) are available at Mendeley Data (doi.org/10.17632/7knhxgvb4s.1). Refer to the original research paper for further discussion [1].

**Table 1:** Control assay biogas pressure and composition.

Elapsed Time (h)	Relative pressure (mBar)	Gas concentration (mmol L^−1^)			
		H_2_	N_2_	CH_4_	CO_2_
0	0	0,0	40,0	0,0	0,0
0,6	27,7	0,0	40,0	0,0	0,0
4,6	40,0	0,0	38,9	0,6	0,3
6,6	19,1	0,0	34,0	0,8	0,4
9,4	23,4	0,0	32,0	1,3	0,4
12,7	20,4	0,0	29,1	1,8	0,6
16,0	46,8	0,0	33,0	3,1	1,3
18,3	110,1	0,0	31,6	5,3	3,7
20,5	98,2	0,0	27,3	7,5	4,7
22,4	64,3	0,0	24,2	8,2	5,3
24,6	110,5	0,0	27,4	12,0	9,0
26,4	190,0	0,0	21,8	12,8	11,5
28,4	502,2	0,3	21,7	17,1	24,7
30,4	307,2	2,9	15,4	15,7	28,9
32,2	161,1	2,8	11,6	12,0	27,3
34,4	101,1	1,7	8,8	10,2	29,7
36,4	70,4	1,6	8,9	11,2	27,3
38,4	61,1	1,7	8,7	11,5	28,8
41,3	68,9	2,2	8,0	10,8	27,9
44,5	68,0	2,8	6,6	10,6	28,5
47,1	55,0	3,1	6,7	9,4	21,7
50,7	54,5	4,1	6,4	9,6	28,0
52,5	38,0	4,4	1,4	10,1	19,6
54,4	28,3	4,8	5,9	10,5	11,3
56,8	47,0	3,7	3,4	6,5	21,9
69,6	200,0	9,0	4,3	8,7	31,2
71,8	78,2	8,1	3,2	6,8	27,7
74,0	64,6	8,7	3,4	6,8	29,1
76,0	63,3	9,7	3,8	5,9	26,8
77,8	67,2	8,2	2,5	4,7	22,9
79,4	63,2	9,6	2,2	4,8	24,6
93,9	800,0	27,0	0,7	3,1	29,6
95,7	12,6	12,6	0,7	1,0	18,2
99,8	16,6	16,6	0,8	1,6	26,4
166,1	0,0	6,8	2,2	1,5	25,5
168,2	0,0	5,1	5,5	0,9	19,8

**Table 2:** Acidic pretreatment assay biogas pressure and composition.

Elapsed Time (h)	Relative pressure (mBar)	Gas concentration (mmol L^−1^)			
		H_2_	N_2_	CH_4_	CO_2_
0	0	0,0	40,0	0,0	0,0
0,9	6,7	0,0	15,2	0,0	0,0
4,8	31,0	0,0	34,1	0,0	0,4
6,8	41,3	0,0	40,6	0,0	1,2
10,2	73,3	1,1	39,8	0,0	3,1
13,0	103,2	3,1	25,9	0,1	4,8
16,2	184,2	6,6	26,2	0,3	7,7
18,3	160,3	8,9	21,2	0,3	9,4
20,7	148,9	11,5	20,2	0,4	12,0
22,7	112,0	11,3	14,9	0,3	11,3
24,7	127,0	15,8	16,2	0,5	15,0
26,5	101,0	15,4	12,9	0,4	12,3
28,5	104,5	17,6	13,1	0,7	16,7
30,6	98,3	20,5	12,7	0,5	18,9
32,4	72,2	18,8	10,7	0,6	18,1
34,5	84,7	28,7	10,2	0,7	18,9
36,5	71,5	19,8	9,1	0,6	19,0
38,5	73,9	20,2	8,4	0,6	19,3
41,5	97,9	20,7	7,5	0,6	19,6
44,6	92,1	22,4	7,4	0,6	21,3
47,2	73,3	21,4	6,4	0,6	20,6
50,8	78,0	17,8	4,6	0,4	19,2
52,8	26,0	17,3	4,4	0,3	19,1
54,6	36,7	18,6	4,5	0,3	20,6
57,9	24,7	13,3	2,6	0,2	15,1
69,7	141,8	10,2	1,0	0,0	10,1
72,0	59,3	13,4	1,8	0,1	15,0
74,1	48,0	22,9	3,8	0,5	23,2
76,1	35,6	20,9	3,9	0,6	22,9
77,9	49,6	17,8	2,3	0,2	19,4
79,5	46,5	14,6	1,5	0,1	16,7
94,0	576,7	11,1	0,0	0,0	12,8
95,8	177,8	0,8	0,0	0,0	0,9
104,1	281,6	27,8	0,8	0,0	29,6
166,3	767,2	24,8	0,0	0,0	30,6
168,4	20,9	21,9	0,0	0,0	28,9

**Table 3:** Thermal pretreatment assay biogas pressure and composition.

Elapsed Time (h)	Relative pressure (mBar)	Gas concentration (mmol L^−1^)			
		H_2_	N_2_	CH_4_	CO_2_
0,0	0	0,0	40,0	0,0	0,0
1,1	37,0	0,0	36,5	0,0	0,7
5,0	6,0	0,0	47,5	0,0	0,6
6,9	10,9	0,0	34,6	0,0	0,6
11,1	13,4	0,0	38,2	0,0	1,0
13,3	10,7	0,0	23,3	0,0	0,5
16,2	23,7	0,2	28,8	0,0	1,2
18,7	18,2	0,2	28,0	0,0	1,5
20,7	20,8	0,5	34,8	0,0	2,9
22,7	36,3	0,6	30,1	0,0	3,8
24,9	47,4	0,8	39,9	0,0	5,2
26,7	24,4	1,0	34,4	0,0	7,1
28,7	23,9	0,6	34,6	0,0	8,2
30,7	32,6	1,6	33,9	0,0	9,4
32,5	33,1	1,7	30,1	0,0	9,3
34,7	42,9	2,2	28,8	0,0	10,3
36,7	52,3	2,7	26,4	0,0	11,2
38,6	48,4	3,0	23,3	0,0	11,9
41,6	83,1	4,9	26,3	0,0	16,0
44,8	107,5	6,9	24,6	0,0	18,1
47,3	110,3	8,5	20,7	0,0	26,1
51,0	153,7	6,4	10,2	0,0	12,0
53,0	93,4	11,2	13,4	0,0	18,1
54,7	109,0	13,3	15,1	0,0	21,4
57,0	118,0	8,4	6,6	0,0	12,3
69,2	445,0	20,8	8,1	0,0	19,8
71,7	149,6	19,1	6,8	0,0	22,7
74,3	104,5	20,3	6,6	0,0	22,2
76,3	84,3	19,9	5,3	0,0	21,4
78,1	71,7	16,5	3,4	0,0	18,2
79,8	60,8	7,8	0,8	0,0	8,4
93,4	331,0	17,9	1,8	0,0	16,1
96,2	109,7	15,6	1,7	0,0	16,5
100,2	77,9	22,6	2,4	0,0	22,4
166,4	1076,6	36,9	0,4	0,0	35,3
168,3	93,2	24,2	0,0	0,0	26,5

**Table 4:** Acidic-thermal pretreatment assay biogas pressure and composition.

Elapsed Time (h)	Relative pressure (mBar)	Gas concentration (mmol L^−1^)			
		H_2_	N_2_	CH_4_	CO_2_
0,0	0	0,0	40,0	0,0	0,0
1,2	47,0	0,0	31,3	0,0	1,3
5,1	21,8	0,0	36,1	0,0	0,8
7,1	12,6	0,0	34,0	0,0	1,2
11,2	26,6	0,1	33,1	0,0	2,0
13,5	24,7	0,2	32,2	0,0	2,9
16,4	42,2	0,7	35,1	0,0	4,7
18,8	42,6	1,0	33,8	0,0	5,7
20,9	42,5	1,3	28,9	0,0	6,4
22,8	36,7	1,5	27,6	0,0	7,1
25,0	48,0	2,3	29,9	0,0	9,2
26,8	36,0	2,8	30,4	0,0	10,5
28,8	45,5	3,1	26,6	0,0	10,6
30,9	43,2	4,0	28,5	0,0	12,6
32,6	40,9	4,2	25,5	0,0	12,6
34,8	46,9	4,3	22,2	0,0	12,3
36,8	42,7	5,5	24,3	0,0	14,7
38,7	41,3	6,1	23,7	0,0	15,9
41,7	47,4	6,6	22,8	0,0	16,9
44,9	48,0	7,0	31,2	0,0	17,5
47,4	35,6	5,9	16,9	0,0	21,8
51,1	33,6	7,0	21,8	0,0	21,1
53,2	28,3	6,1	16,8	0,0	17,4
54,9	28,0	5,4	14,6	0,0	16,1
57,2	34,2	6,9	17,6	0,0	19,4
70,3	131,2	7,7	12,3	0,0	18,2
72,2	65,9	8,3	12,9	0,0	20,6
74,4	56,0	10,6	13,8	0,0	22,6
76,4	62,1	10,5	11,6	0,0	20,9
78,2	66,0	5,9	5,3	0,0	11,8
79,9	48,5	10,6	9,4	0,0	19,4
93,5	611,1	24,6	6,2	0,0	26,8
96,3	268,3	12,0	1,2	0,0	14,6
100,3	291,8	28,4	3,7	0,0	10,9
166,5	320,2	18,3	0,8	0,0	25,8
168,6	8,3	17,8	0,9	0,0	25,8

**Table 5:** Thermal-acidic pretreatment assay biogas pressure and composition.

Elapsed Time (h)	Relative pressure (mBar)	Gas concentration (mmol L^−1^)			
		H_2_	N_2_	CH_4_	CO_2_
0,0	0	0,0	40,0	0,0	0,0
1,3	36,0	0,0	32,5	0,0	1,3
5,2	15,0	0,0	36,8	0,0	0,7
7,2	9,8	0,0	36,0	0,0	1,2
11,3	22,6	0,0	39,5	0,0	2,3
13,6	16,2	0,0	31,8	0,0	2,2
16,6	42,5	0,0	31,2	0,0	3,1
18,8	38,3	0,2	31,4	0,0	4,4
21,0	46,5	0,7	30,3	0,0	5,6
23,0	45,8	1,2	29,0	0,0	6,6
25,2	98,3	2,2	29,3	0,0	8,3
27,0	34,6	3,0	27,8	0,0	9,4
29,0	47,5	4,2	29,5	0,0	11,3
31,0	61,9	5,0	27,0	0,0	11,8
32,8	53,3	5,8	26,1	0,0	12,9
34,9	55,4	6,2	22,4	0,0	12,6
36,9	50,7	7,7	23,3	0,0	14,4
38,9	50,2	7,5	20,3	0,0	13,9
41,9	52,6	9,2	22,1	0,0	16,3
45,1	51,8	9,4	20,5	0,0	16,5
47,6	27,1	9,2	19,3	0,0	16,7
51,2	21,4	8,5	20,3	0,0	18,6
53,2	23,7	1,1	1,5	0,0	2,3
55,0	2,0	2,3	4,3	0,0	5,1
57,3	8,5	7,2	16,3	0,0	16,3
70,5	14,1	7,1	16,9	0,0	17,2
72,3	11,3	7,0	17,4	0,0	17,6
74,5	14,3	7,8	17,4	0,0	18,4
76,5	13,9	3,1	7,5	0,0	7,5
78,3	12,5	6,4	14,2	0,0	15,6
79,7	6,1	7,1	18,5	0,0	19,9
93,7	118,3	10,0	15,5	0,0	19,5
96,5	65,0	9,3	10,7	0,0	14,9
100,5	85,6	12,0	12,5	0,0	18,9
166,7	745,5	19,4	9,1	0,0	35,8
168,8	63,6	12,6	5,8	0,0	27,8

**Fig. 1:**
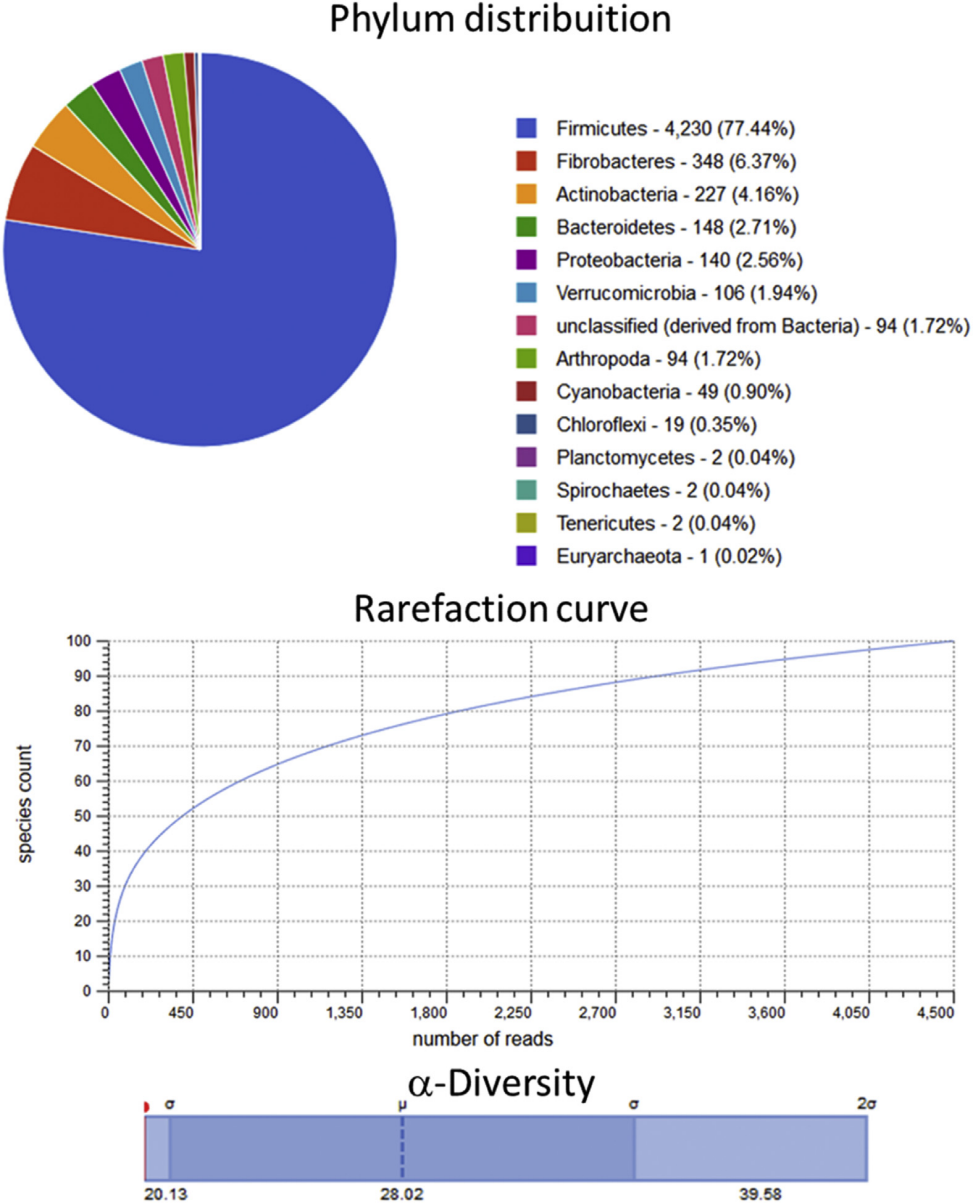
Control assay microbiological diversity.

**Fig. 2:**
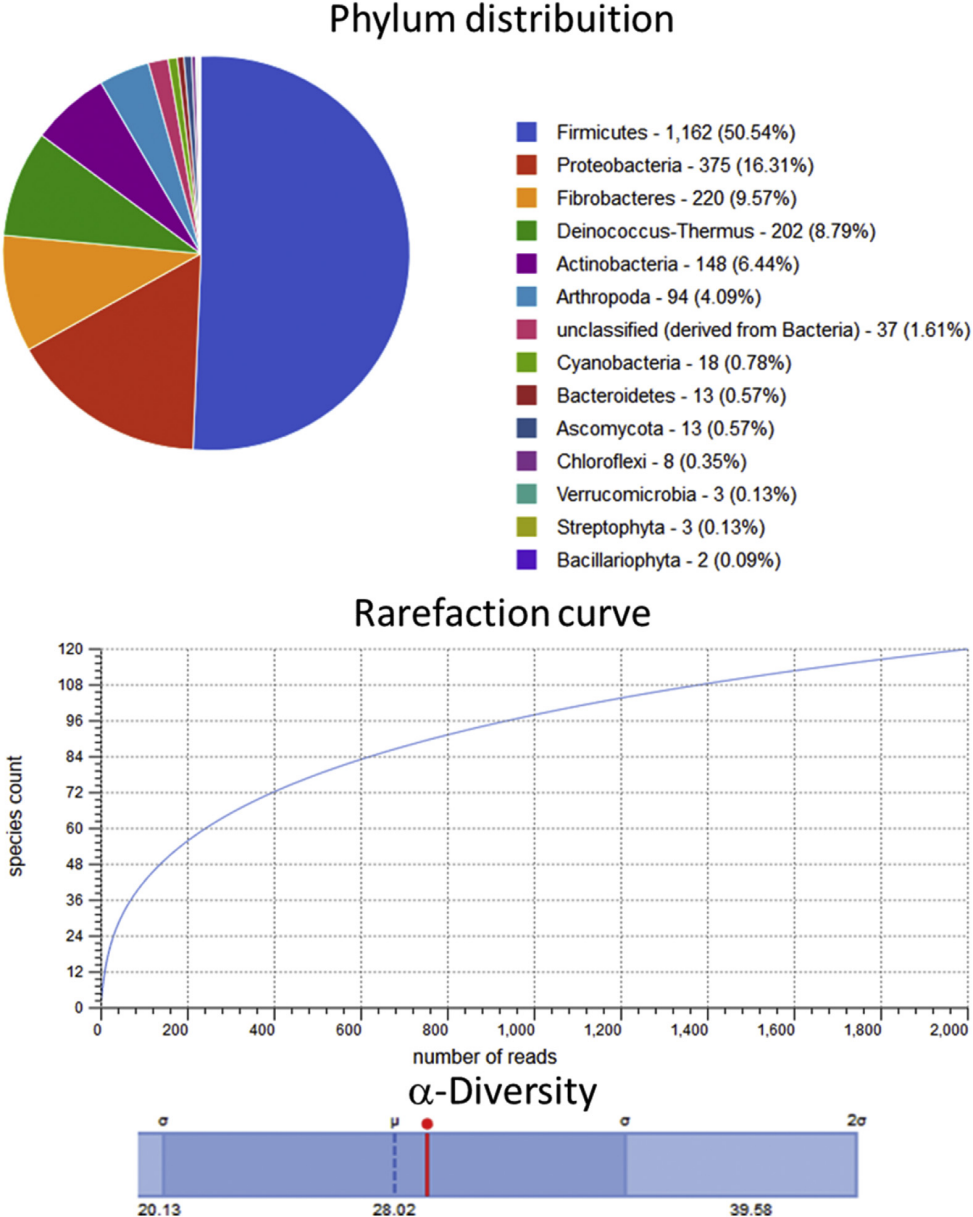
Acidic pretreatment assay microbiological diversity.

**Fig. 3:**
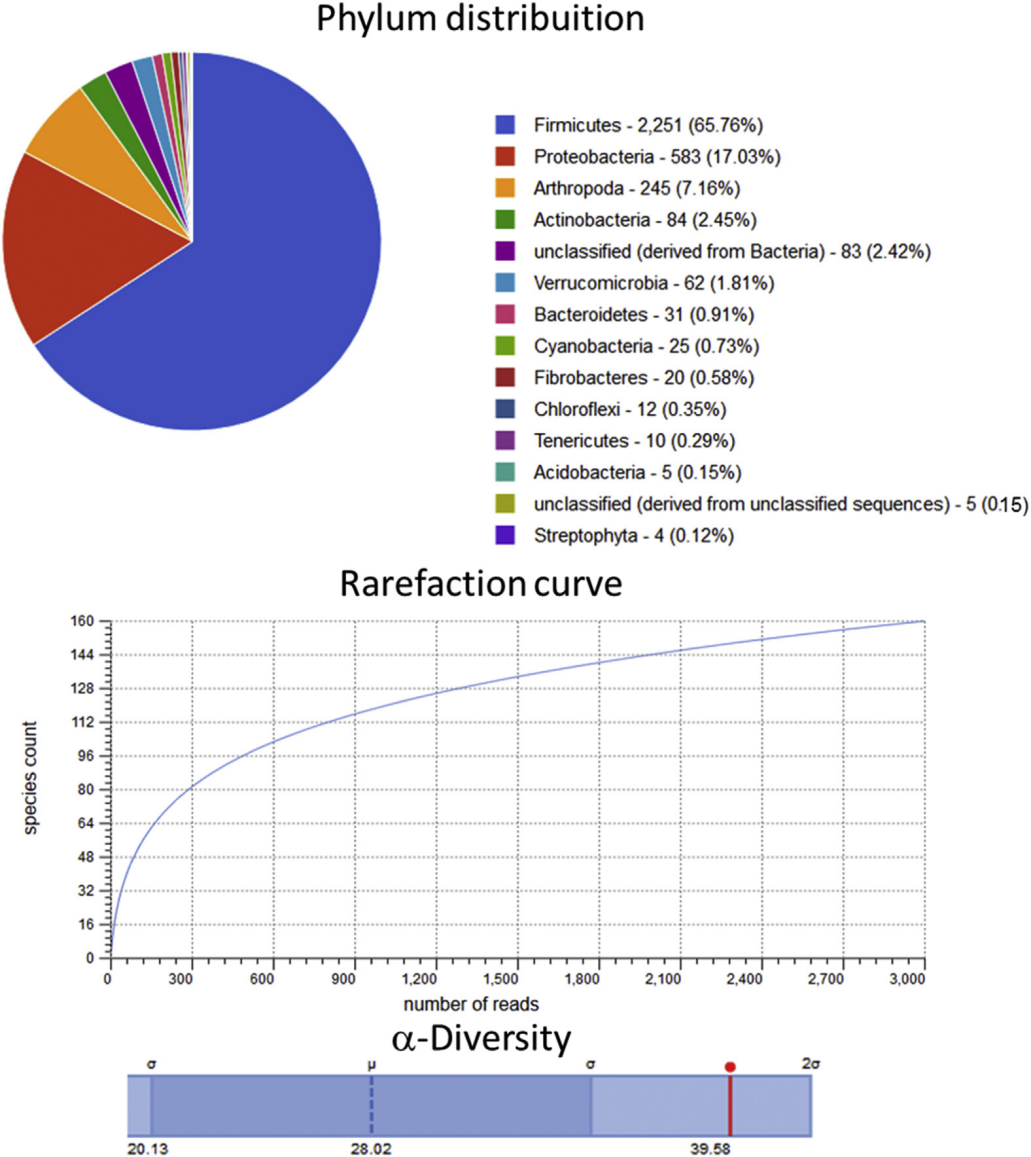
Thermal pretreatment assay microbiological diversity.

**Fig. 4:**
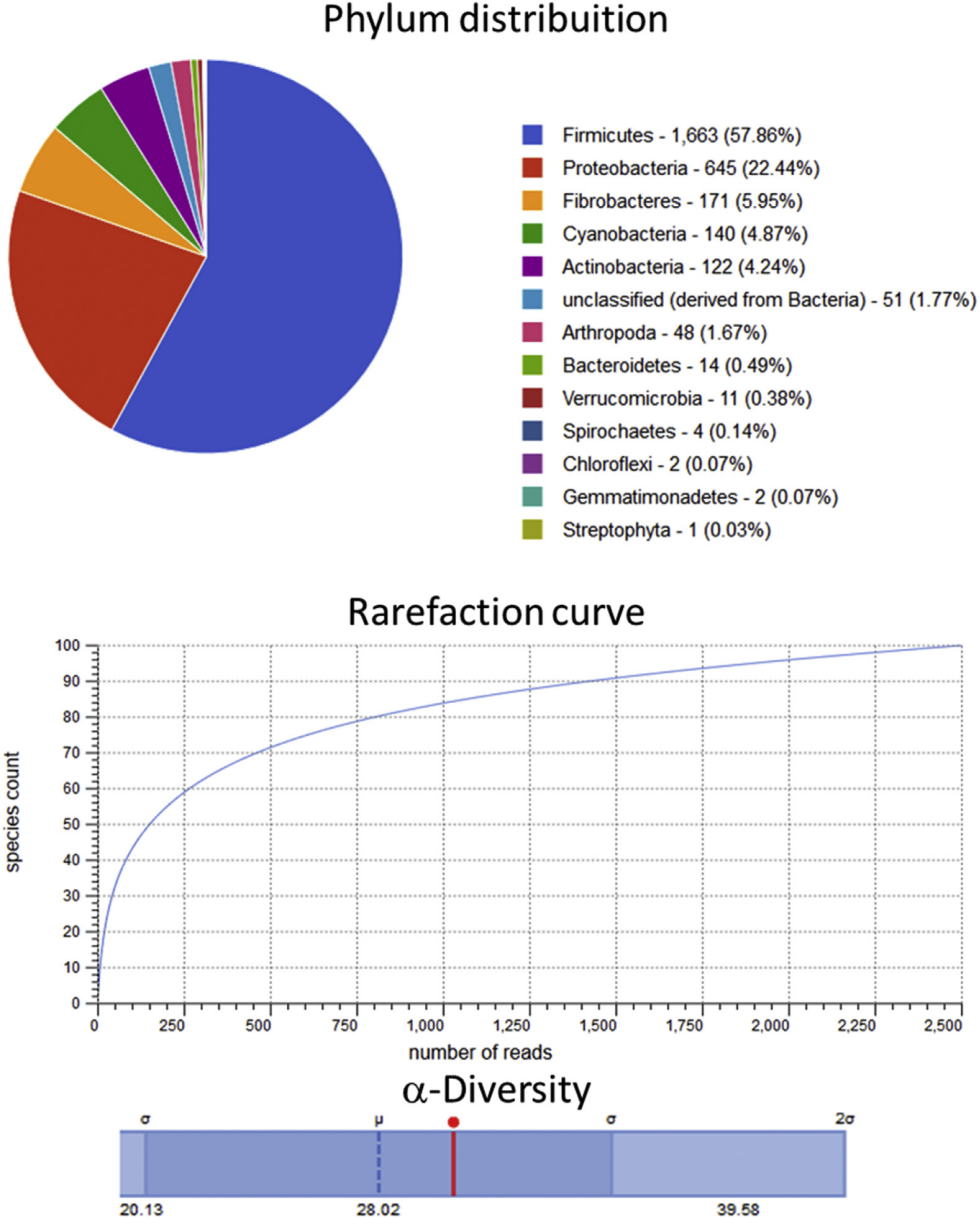
Acidic-thermal pretreatment assay microbiological diversity.

**Fig. 5:**
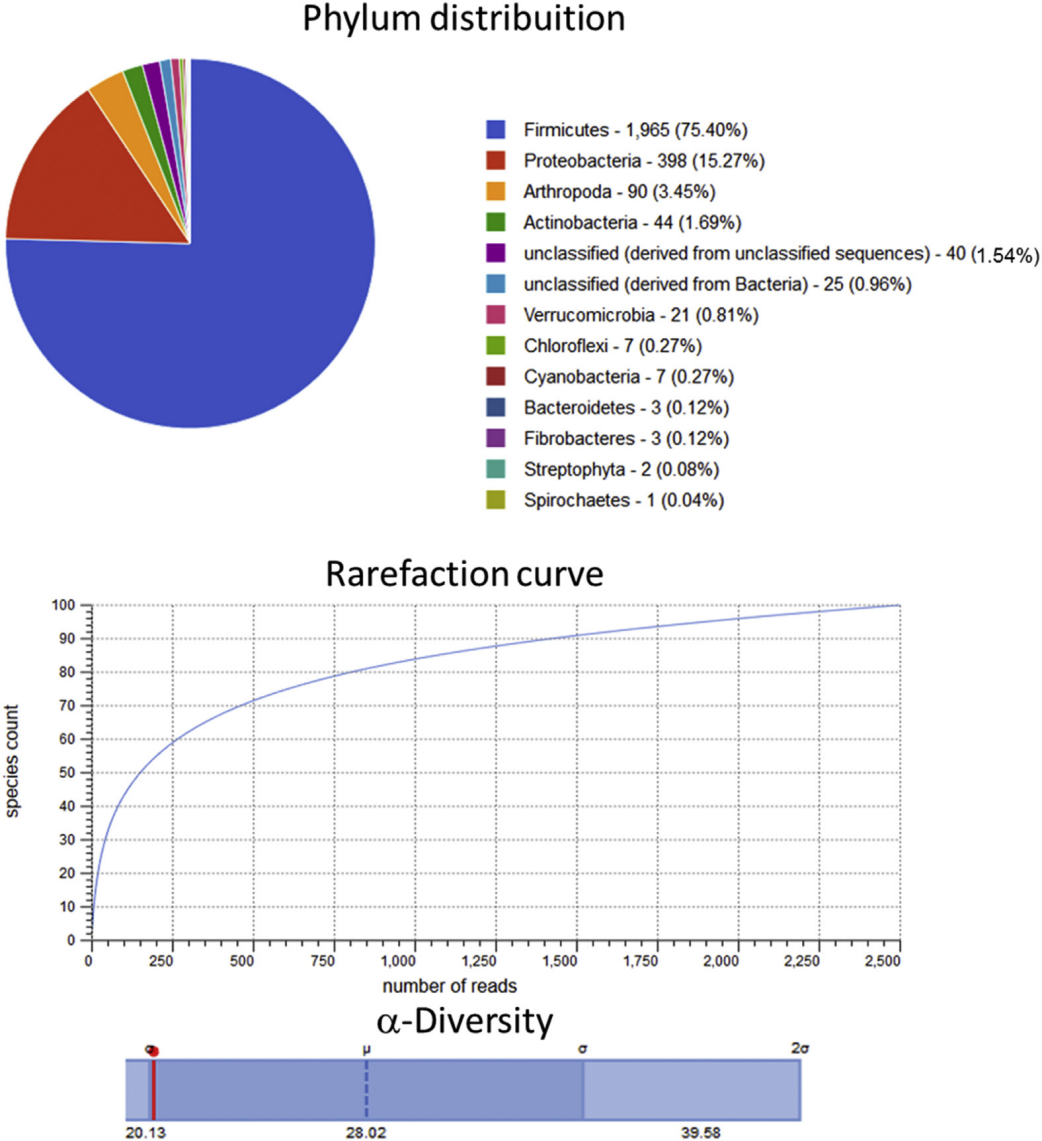
Thermal-acidic pretreatment assay microbiological diversity.

Raw metagenomic data included only phylum level, rarefaction curve and α-diversity (shown as a red line in each graph) is depicted for the control assay (Fig. 1), acidic pretreatment (Fig. 2), thermal (Fig. 3), acidic-thermal (Fig. 4), and thermal-acidic (Fig. 5).

## 2 Experimental design, materials, and methods

### 2.1 Experimental set-up

All assays used xylose as carbon source. Media was supplemented with nutrients [2], and urea was used as a nitrogen source [3]. pH of the medium was corrected to 6.5. All experiments were performed in single batches in Duran™ flasks. Initially, the headspace was replaced by nitrogen to avoid any trace of oxygen. In all assays, the initial inoculum concentration was 6.6 ± 0.3 g total volatile solids (TVS) L^−1^. The original inoculum was obtained from a UASB reactor treating poultry slaughterhouse wastewater. All experiments were carried out under mesophilic conditions (30 °C) in a shaker incubator with orbital stirring at 150 RPM.

### 2.2 Biogas sampling and analyzes

The pressure was measured with a digital manometer. Afterward, gas samples were withdrawn with a syringe with a stopper for injection for gas chromatography to determine the concentration of H_2_, N_2_, CH_4_, and CO_2_ in the biogas [2]. After the pressure measurement and samples collecting, the flask headspace was depressurized until it reaches the atmospheric pressure to avoid excessive CO_2_ solubilization in the liquid phase, which may interfere in autotrophic processes.

### 2.3 Sample metagenomics

Samples of the inoculum were taken after all experiments end. Genomic DNA was extracted and purified. Amplification of 16S rRNA genes used a primer set targeting a conserved region (16S rRNA) [4]. The amplicons were sequenced with a 454 Genome Sequencer FLX (Roche). Sequences were processed through the procedures established for environmental samples [5], [6]. Sequences were annotated using the Metagenomic Rapid Annotations using Subsystems Technology (MG-RAST) [7].
